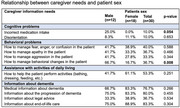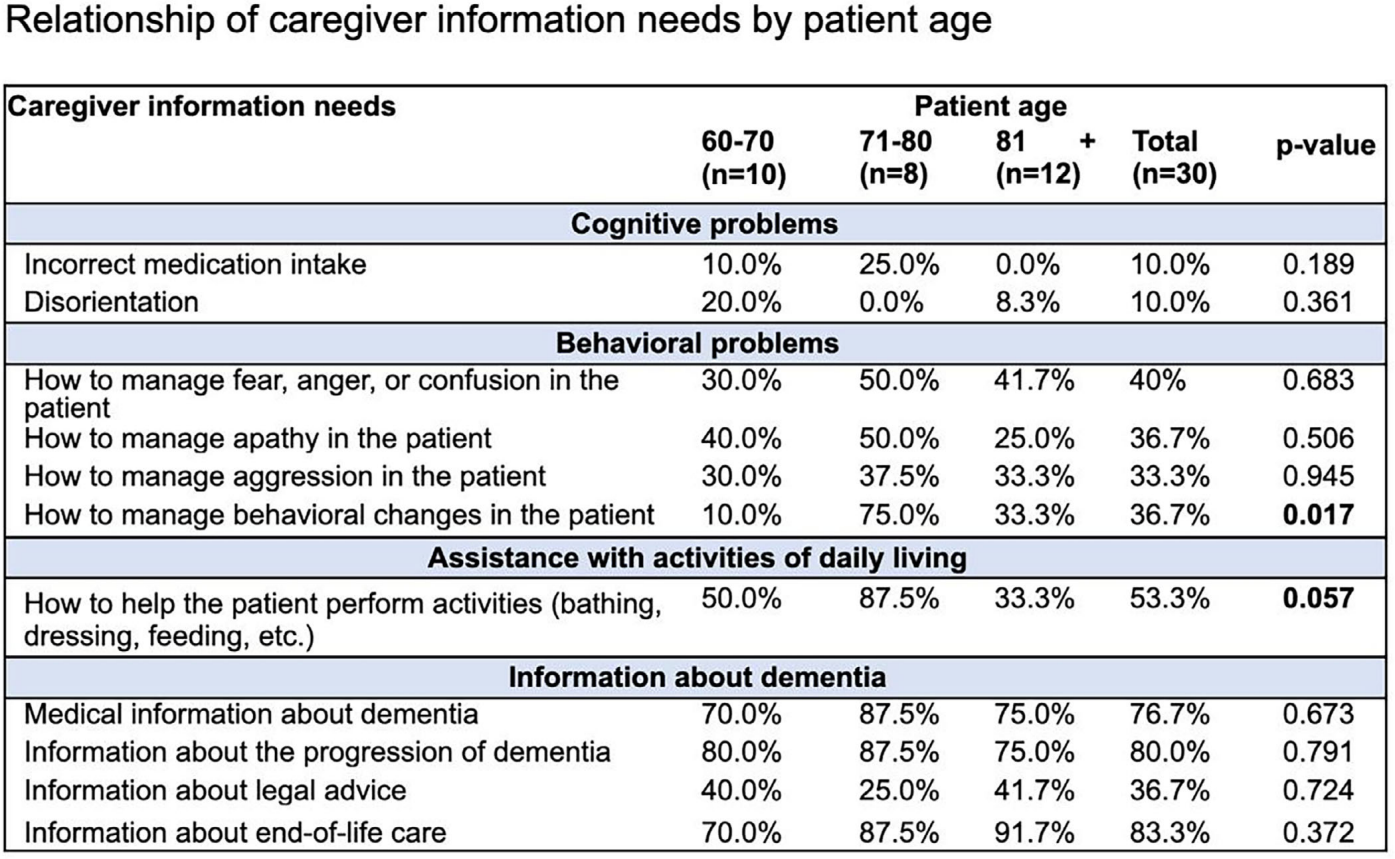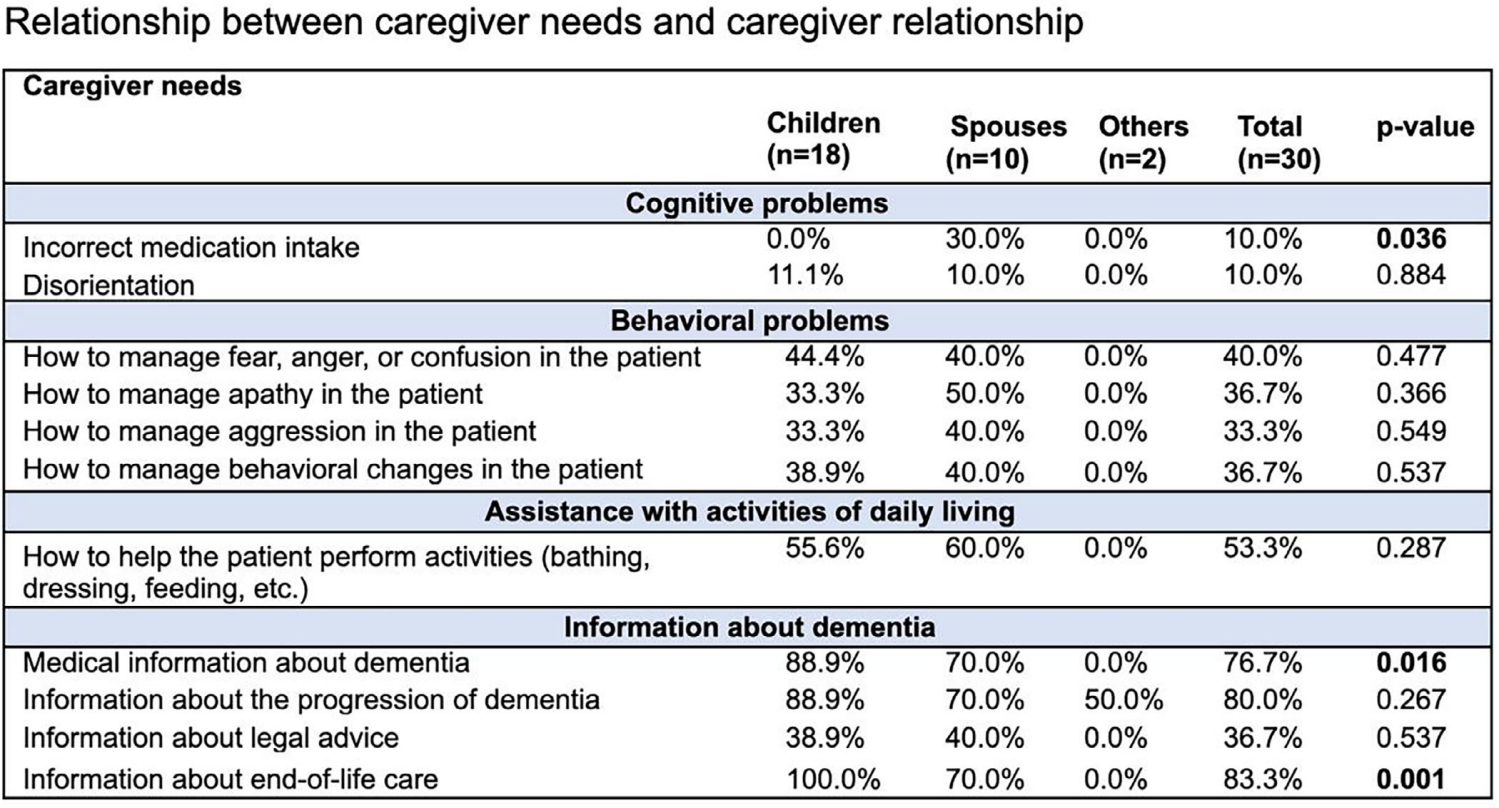# Assessment of the Needs in Caregivers of People with Advanced Dementia

**DOI:** 10.1002/alz70858_104198

**Published:** 2025-12-26

**Authors:** Luis Raul Montiel‐Velazquez, Sandra Lizeth Celaya‐Hernández, Mariana Longoria Ibarrola, Ana Luisa Sosa, Isaac Acosta‐Castillo

**Affiliations:** ^1^ National Institute of Neurology and Neurosurgery, Tlalpan, DF, Mexico; ^2^ National Institute of Neurology and Neurosurgery, Mexico City, Mexico; ^3^ Instituto Nacional de Neurología y Neurocirugía, Mexico City, DF, Mexico

## Abstract

**Background:**

Advanced dementia represents a challenge marked by significant cognitive deterioration that impacts an individual's ability to recognize family, communicate effectively, and maintain independence. In this context, it's essential to identify the unresolved needs of the caregiver to optimize functionality and care and improve the quality of life for both the patient and the caregiver.

**Method:**

A cross‐sectional study was conducted with 30 caregivers of patients with advanced dementia who attended the Cognitive Aging and Dementia clinic at the National Institute of Neurology and Neurosurgery, between February and April 2024. Participants completed a sociodemographic questionnaire and a care needs assessment questionnaire, in addition to some questions specifically developed for this population by the research team.

**Result:**

We obtained 30 responses from primary caregivers of patients with advanced dementia, mostly women and children (80% and 60%, respectively). Regarding the characteristics of patients with advanced dementia, the majority were female (60%) and over 80 years old (40%). When analyzing caregivers' needs based on the patient's sex, a slight significance was observed in the incorrect administration of medications (10%, *p* = 0.054) and in the need for information on managing behavioral changes (36.7%, *p* = 0.008). In relation to the patient's age, a statistically significant association was found between caregiver needs, particularly in managing behavioral changes in patients aged 71‐80 years (75%, *p* = 0.017), as well as the need for information on how to perform activities of daily living (87.5%, *p* = 0.057). Regarding the relationship between caregiver needs and behavioral problems, these needs were more frequent among female caregivers (45.8%), with a significant result (*p* = 0.046). Concerning primary caregivers' needs related to behavioral changes and assistance with activities of daily living, no significance was found. However, the need for information about end‐of‐life care showed a significant result (*p* = 0.001), with 100% of children reporting this need.

**Conclusion:**

The implementation of programs that provide specific support for the caregiver's needs is required, particularly regarding the management of behavioral changes, activities of daily living, dementia progression, and end‐of‐life care.